# Gradual onset and recovery of the Younger Dryas abrupt climate event in the tropics

**DOI:** 10.1038/ncomms9061

**Published:** 2015-09-02

**Authors:** J.W. Partin, T.M. Quinn, C.-C. Shen, Y. Okumura, M.B. Cardenas, F.P. Siringan, J.L. Banner, K. Lin, H.-M. Hu, F.W. Taylor

**Affiliations:** 1 Institute for Geophysics, Jackson School of Geosciences, University of Texas at Austin, Austin, Texas 78758, USA; 2 Department of Geological Sciences, Jackson School of Geosciences, University of Texas at Austin, Austin, Texas 78712, USA; 3 High-Precision Mass Spectrometry and Environment Change Laboratory (HISPEC), National Taiwan University, Taipei 10617, Taiwan, ROC; 4 Marine Science Institute, University of the Philippines-Diliman, Quezon City 1101, Philippines

## Abstract

Proxy records of temperature from the Atlantic clearly show that the Younger Dryas was an abrupt climate change event during the last deglaciation, but records of hydroclimate are underutilized in defining the event. Here we combine a new hydroclimate record from Palawan, Philippines, in the tropical Pacific, with previously published records to highlight a difference between hydroclimate and temperature responses to the Younger Dryas. Although the onset and termination are synchronous across the records, tropical hydroclimate changes are more gradual (>100 years) than the abrupt (10–100 years) temperature changes in the northern Atlantic Ocean. The abrupt recovery of Greenland temperatures likely reflects changes in regional sea ice extent. Proxy data and transient climate model simulations support the hypothesis that freshwater forced a reduction in the Atlantic meridional overturning circulation, thereby causing the Younger Dryas. However, changes in ocean overturning may not produce the same effects globally as in Greenland.

## Abstract (web summary)

The Younger Dryas cooling event has been identified in ice records in the Northern Hemisphere, but the effects of this cold snap on the tropics are poorly known. Here, the authors present a speleothem record and model simulations, showing that tropical hydroclimate recovered slower than temperatures in Greenland.

## Graphical abstract

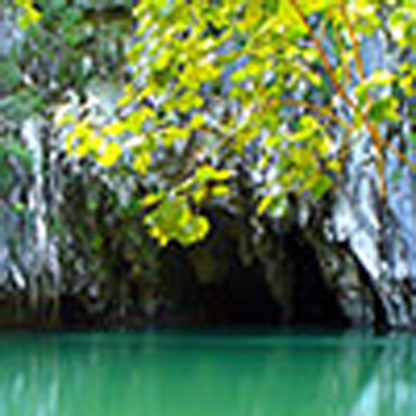

The Younger Dryas (YD) was an abrupt climate change event that affected climate across much of the Earth approximately 12.5 kyr ago (before 1950 CE, hereafter^1^). An abrupt climate event is operationally defined as ‘one that takes place so rapidly and unexpectedly that human or natural systems have difficulty adapting to it'^2^. Greenland ice core records suggest that the onset of the YD occurred rapidly in possibly as little as 3 years and that the termination occurred over ∼60 years^3^. The prevailing hypothesis for the cause of the YD is a sudden influx of freshwater into the North Atlantic and the attendant weakening of the Atlantic meridional overturning circulation (AMOC)^4^,^5^,^6^,^7^.

Greenland ice core records, particularly δ^18^O data (a proxy for annual temperature and moisture source^1^), have commonly been used as the record of reference when discussing the YD due to the large signal in the proxy records, Greenland's location in the North Atlantic and the small dating uncertainties of ±120 years (2*σ*) in the layer counting of young ice (∼12 kyr ago)^8^. Although temperature provides an important constraint on the energetics of the climate system, characterizing hydroclimate change is equally important because changes in rainfall can have large, sometimes devastating, socio-economic impacts. Cave formations (for example, stalagmites) have emerged as a valuable proxy archive for investigating hydroclimate, and are particularly applicable for studying abrupt climate change events due to the high precision associated with the absolute U–Th dating of these archives^9^,^10^.

In the present paper, a new hydroclimate record from the deep tropics is combined with previously published hydroclimate and temperature records to better understand how a climate change event such as the YD evolves. Our conclusions hold important implications for the occurrence and prediction of future climate events.

## Results

### The Younger Dryas signal in the Western Pacific

**Figure 1:**
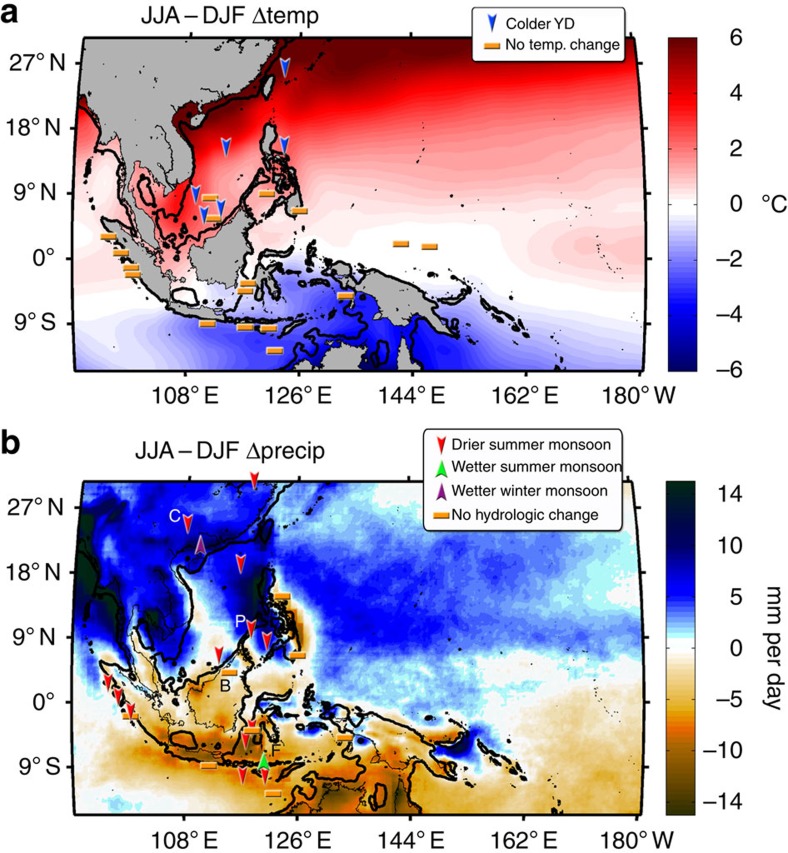
Synthesis of published paleoclimate data during the Younger Dryas from the western Pacific. Seasonal differences (JJA minus DJF) in (**a**) SST (°C; 1998–2012 (ref. 65)) and (**b**) precipitation (mm h^−1^; 1998–2012 (ref. 66)) in the western tropical Pacific. **(a)** The symbols indicate the temperature signal in proxy data during the YD (Supplementary Table 1). The blue arrows denote cooling of 1 °C or less. The darker line is the coast at ∼12 kyr ago, that is, the isobath at 60 m below modern sea level. **b** The symbols indicate the hydroclimate signal in proxy data during the YD (Supplementary Table 1). Letters denote the locations of sites discussed: C, China, P, Palawan, B, Borneo and F, Flores. Both temperature and precipitation proxy data indicate that cooling and drying were restricted to the areas of the Asian summer monsoon.

The YD event is captured in select stalagmite records, including those from China^11^, India^12^, Brazil^13^ and the United States^14^, but few stalagmite records exist from the deep tropics. This paucity of rainfall records is especially true in caves on islands in the Western Pacific Warm Pool^15^,^16^. The Western Pacific Warm Pool represents a major source of heat and water vapour in the global climate system, and its changes affect climate variability across the globe on a multitude of timescales. Paleoclimate records from in and around the western tropical Pacific show minimal temperature responses during the YD^17^,^18^,^19^,^20^,^21^,^22^,^23^,^24^,^25^,^26^,^27^,^28^, with slight cooling confined to the areas around mainland Asia^29^,^30^,^31^,^32^,^33^,^34^ (Fig. 1a, Supplementary Table 1).

The hydroclimate response to the YD in the Western tropical Pacific and adjacent regions was spatially and seasonally variable (Fig. 1b): the northern summer monsoon weakened, as suggested by δ^18^O_sw_ marine sediment records (a proxy for salinity) from the South China Sea^29^,^30^,^33^, Sulu Sea^17^ and eastern Indian Ocean^20^,^23^ and stalagmite records from southeastern China^35^ (with a hint of a concomitant increase in the boreal winter and austral summer monsoons in China^36^ and Flores^16^,^37^, respectively). In contrast, most proxy records from around Borneo^15^,^38^, the Banda Sea^19^, the Timor Sea^24^ and east of the Philippines^19^,^31^, where the primary rainy season occurs during boreal winter, show no hydroclimate response to the YD, with a few exceptions^25^,^26^,^27^,^28^ that are closely tied to the Indonesian throughflow. These records suggest that the YD primarily weakened the boreal summer monsoon in the western tropical Pacific and may have slightly strengthened the austral summer monsoon. A recent study^39^, however, argues that the stalagmite records from southeastern China may reflect isotopic changes in precipitation over India via atmospheric moisture transport and not local hydroclimate, but the records would still indicate a reduction in the boreal summer monsoon. To our knowledge, there is no direct evidence of boreal summer monsoon changes in the Western tropical Pacific to date.

### Stalagmite δ^18^O record from Palawan, western Philippines

Here we present a stalagmite δ^18^O record of hydroclimate variability from the Puerto Princesa Subterranean River National Park in Palawan, Philippines (10.2° N and 118.9° E), to better understand the precipitation response of the Western tropical Pacific during the YD. The rainy season today in Palawan (May–November) contributes ∼80% of the total annual rainfall of ∼2,000 mm, and the seasonal variation in average temperature is small (27–29 °C). The limited seasonal variation in temperature and the high humidity of tropical cave environments suggest that changes in rainfall δ^18^O values dominate the stalagmite δ^18^O signal in Palawan^40^. The stalagmite δ^18^O signal most likely records changes in wet season rainfall amount via the ‘amount effect,' which is an empirical relationship between rainfall amount and rainfall δ^18^O values in the tropics^41^. Studies at similar sites in the Western tropical Pacific^40^,^42^ show an inverse relationship between rainfall amount and rainfall δ^18^O composition: a change of ∼1,000 mm of rainfall per year results in a 1‰ change in rainfall (that is, stalagmite) δ^18^O values on suborbital timescales (decades to centuries) when ocean basin water transfer is minimal^43^.

The bottom of the stalagmite is U–Th dated to ∼13.7 kyr ago, and a hiatus in growth constrains the youngest part of the record to ∼10.8 kyr ago (Supplementary Fig. 1). The age model for the stalagmite δ^18^O record consists of 12 U–Th dates with an average precision of ±78 years (2*σ*; Methods; Supplementary Table 2; Supplementary Fig. 2). Subsamples for δ^18^O analysis were drilled every 1 μm per subsample for the upper 2 cm and 0.5 μm for the lower 16.5 cm of the stalagmite, resulting in an average temporal resolution of ∼7 years per δ^18^O data point. We assessed whether the stalagmite formed in isotopic equilibrium with cave dripwater and conclude that non-equilibrium effects on the stalagmite isotopic composition are limited (Supplementary Figs 3 and 4).

**Figure 2:**
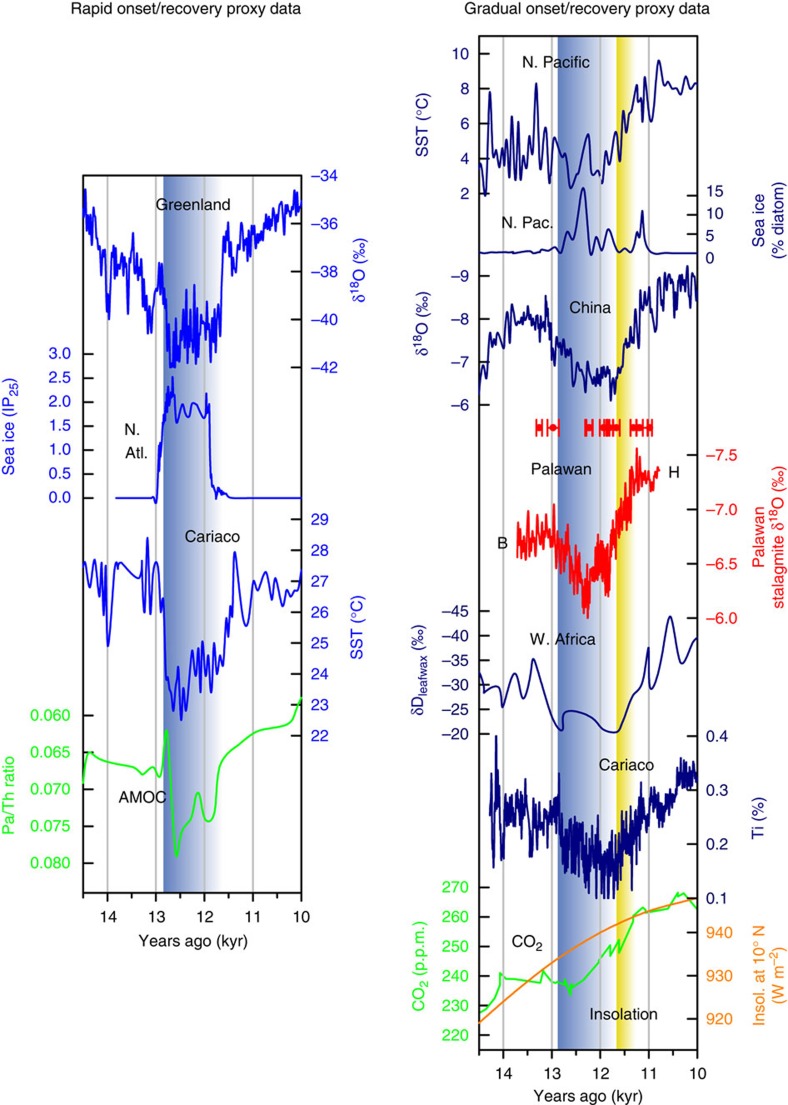
Proxy data for temperature, hydroclimate and ocean circulation highlighting the timing and magnitude of the YD. The blue shaded bars indicate the timing of the canonical 12.8–11.6 kyr ago YD derived from Greenland temperatures. The yellow shading indicates a later, more gradual end to the YD ∼300 years later based on proxy time series of tropical hydroclimate. The red data points with error bars are the U–Th dates for the new stalagmite record from Palawan, Western Philippines. The timing of the blue bar is more coeval with Greenland temperature^8^ and Cariaco Basin SST^47^, which respond to the AMOC^7^ and sea ice^46^, whereas the yellow bar timing is seen in proxy records outside the northern North Atlantic^35^,^48^,^49^,^50^ and is more concurrent with the timing of increases in NH summer insolation^67^ and CO_2_ (ref. 68).

**Figure 3:**
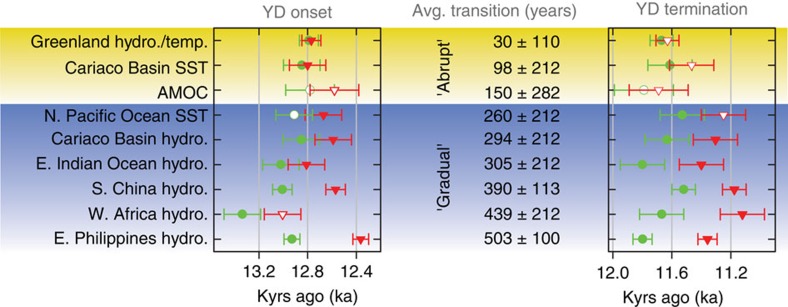
The timing of the onset and termination of the YD in well-dated paleoclimate proxy time series. The initiation (green circles) and completion (red triangles) of both the onset and the termination are depicted. Filled symbols indicate that the timing is calculated using a Bayesian change point method^69^. When the method did not detect a change point, visual determinations were used (open symbols). Error bars on the timing come from the age uncertainty reported in the published record. The exception to this is the Palawan record, for which the error bars are derived from the width of the distributions in Supplementary Fig. 5. All time series show a contemporaneous initiation of the onset and the termination, as has been established in previous studies. However, the completion of both parts of the YD in hydroclimate and temperature proxies outside of the North Atlantic occurs more gradually and does not fully recover until at least 300 years after Greenland temperatures.

The Palawan stalagmite δ^18^O record (Fig. 2, red line) shows an increase during the YD (12.9–11.7 kyr ago, Greenland YD chronology^8^; Fig. 2, blue shading), reflecting drier conditions. The initiation of the YD event, represented by a decrease in stalagmite δ^18^O values of 0.5‰ (decadal-scale shifts of ∼0.5 m per year or 1.4 mm per day), occurs at 12.92±0.08 kyr ago (Supplementary Fig. 5), which is not significantly different from the cooling in Greenland at 12.79±0.08 kyr ago (Fig. 3). In addition, the timing of the initiation of the recovery in Palawan at 11.79±0.08 kyr ago matches the warming in Greenland at 11.67±0.07 kyr ago. However, those events represent the extent of the similarities in timing, as the initiation and recovery of the YD in Greenland are more abrupt than in Palawan. Indeed, the final recovery of the termination in Palawan occurs at 11.36±0.08 kyr ago (Supplementary Fig. 5), which is significantly later than the timing of the full recovery in Greenland at 11.63±0.08 kyr ago.

### Comparison of temperature and hydroclimate proxy records

The timing of the initiation of the onset and termination of the YD are contemporaneous among the various proxy records in the Atlantic and Pacific sectors, suggesting a common driver: the AMOC (Fig. 2). At the onset of the YD, the North Atlantic sea surface temperature (SST)^44^,^45^ and Greenland temperature^1^ decreased as the AMOC weakened^7^ and sea ice extent increased rapidly^46^. SSTs in the Cariaco Basin off the coast of Venezuela in the Caribbean Sea also decreased abruptly^47^. Cooling in the North Atlantic reduced the meridional SST gradient in the tropical Atlantic and eventually shifted the intertropical convergence zone (ITCZ) southward^48^, resulting in decreased rainfall over the Cariaco Basin^48^ and western Africa^49^. In the North Pacific, an increase in sea ice accompanied lower SSTs^50^. During the period of reduced AMOC, the northern summer monsoons in China^11^ and the western tropical Pacific (this study) weakened. Sea surface salinity in select locations around the western Pacific also exhibited a change towards more saline conditions^17^,^20^,^25^,^26^,^27^,^28^,^33^.

Although proxy data agree on how the YD affected climate, data on the temporal evolution of the YD fall into two categories: gradual (duration of >100 years to reach and recover from full YD conditions) or abrupt (duration of 10–100 years to reach and recover from full YD conditions). These timings are based on the adaptation time of human and natural systems to drastic changes in climate^2^. The rapid resumption of the AMOC (∼100 years), coupled with the retreat of insulating sea ice at ∼11.6 kyr ago^46^, likely led to the abrupt warming (40 years to recover from full YD conditions) in Greenland surface air temperature^51^,^52^. SSTs from a well-dated marine sediment core from the Cariaco Basin in the Caribbean Sea show the same rapid onset (48 years to reach full YD conditions) and termination (147 years to recover from full YD conditions) of the YD^47^. However, a proxy record of hydroclimate from the same basin^48^ shows a much more gradual onset (261 years) and termination (327 years), such that the completion of the termination occurred at 11.30±0.15 kyr ago (Fig. 2). Well-dated hydroclimate records from several tropical locations also show a gradual YD response. In addition, rainfall in western Africa^49^ displays a gradual onset (331 years) and termination (547 years), with completion of the termination occurring at 11.12±0.15 kyr ago. A gradual onset and termination also occurred in the northern summer monsoons in China^11^ (onset: 437 years; termination: 343 years; completion of termination: 11.18±0.08 kyr ago), in the Western tropical Pacific (this study; onset: 565 years; termination: 440 years), and in the sea surface hydroclimate conditions in the eastern Indian Ocean^20^ (onset: 210 years; termination: 400 years; completion of termination: 11.40±0.15 kyr ago). A gradual onset (240 years) and termination (280 years) are also observed in a well-dated SST record from outside of the tropics in the North Pacific (completion of termination: 11.25±0.15 kyr ago, at which time the local sea ice also disappeared) (Fig. 2). The time required to reach and recover from full YD conditions in the tropical hydroclimate records and temperature in the North Pacific (gradual; Fig. 3) clearly differs, beyond the range of error, from that of temperature in the Atlantic, sea ice in the North Atlantic, and the AMOC (abrupt; Fig. 3).

### YD in transient climate model simulations

**Figure 4:**
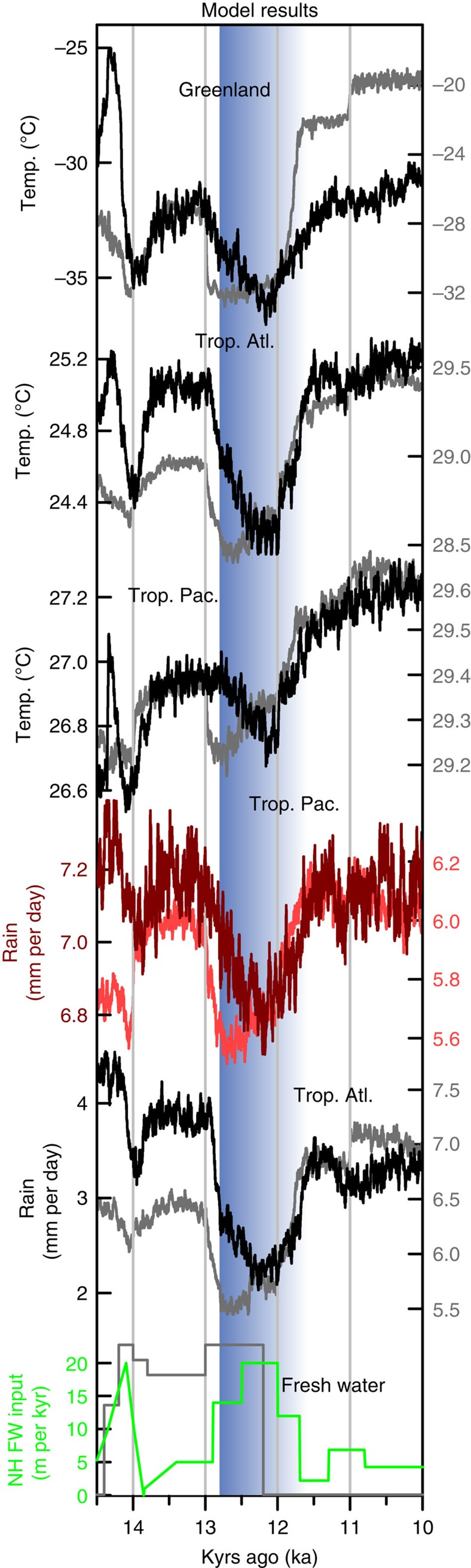
Climate model output highlighting the timing and magnitude of the YD. The blue shaded bars indicate the timing of the canonical YD from 12.8 to 11.6 kyr ago. Climate model outputs from the CCSM3 TraCE-21ka^53^ (black curves) and LOVECLIM DGNS^54^ (grey curves; secondary y-axis has the same units as the primary y-axis) experiments are averaged over Greenland (annual mean; 65–75° N, 50–30° W), the tropical North Atlantic (JJA; 5–15° N, 70–20° W), and the tropical Indo-Pacific (JJA; 5–15° N, 80–140° E) to approximate the locations of the proxy records in Fig. 2. The model data are smoothed with a 21-year running mean filter. NH freshwater forcing in the two model simulations is shown in the bottom panel.

We compare the proxy records with the deglacial climate transition simulated by two climate models of different complexities (CCSM3 (ref. 53) and LOVECLIM^54^; see Methods). These climate models are forced with temporally varying greenhouse gas concentrations, insolation, ice-sheet topography and meltwater fluxes from the last glacial maximum (19–21 kyr ago) through the deglaciation. The temporal evolution of climate simulated by these models qualitatively agrees with the proxy records, indicating that cooling in the North Atlantic led to cooling and drying in the northern tropical Atlantic and Indo-Pacific (Fig. 4). The timing of the simulated YD closely follows the evolution of prescribed meltwater influx into the North Atlantic, which supports the hypothesis that this climate event was mediated by changes in the AMOC. The magnitude of SST change in the tropical Indo-Pacific is <0.5 °C and agrees with the uncertainty of paleoclimate reconstructions of SST^25^,^27^. The magnitude of precipitation change in the tropical Indo-Pacific is, however, substantially smaller than the amount suggested by the new stalagmite proxy data from Palawan (Fig. 2; Supplementary Fig. 7). The underestimated hydroclimate response in the tropical Indo-Pacific may be attributed to several factors, although we are not able to pinpoint the exact cause: the comparison of grid-scale model output to proxy data at a specific grid point, the relatively low horizontal resolutions of the atmospheric component models (3.75° in CCSM3 and 5.63° in LOVECLIM), uncertainty in the meltwater forcing, and/or uncertainty in the conversion from stalagmite δ^18^O values to rainfall amount.

**Figure 5:**
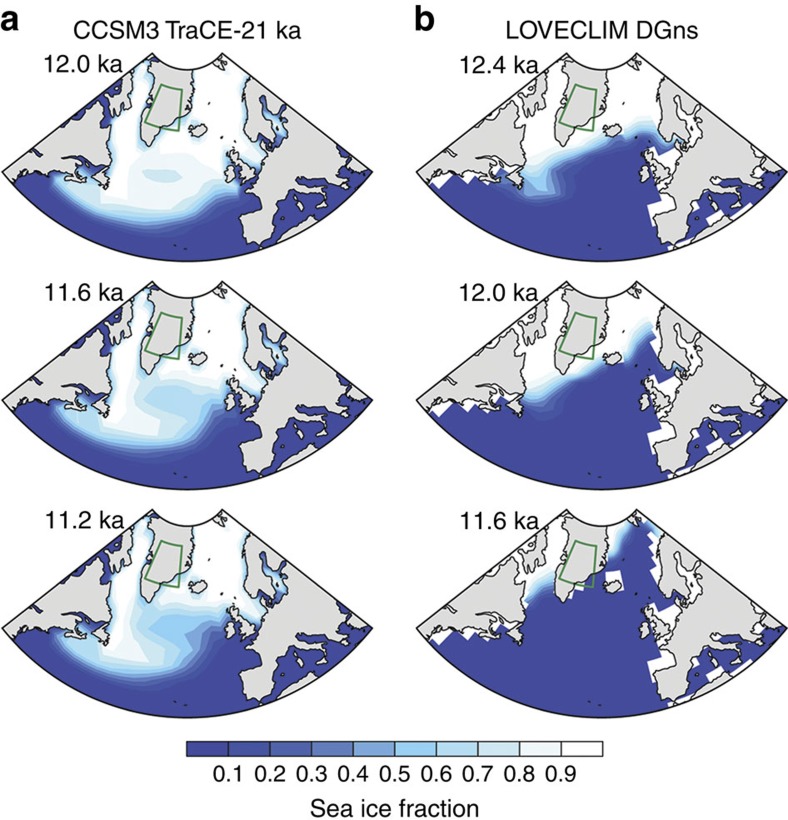
Sea ice coverage in the North Atlantic during the YD from two climate models. Output is from CCSM3 (**a**) and LOVECLIM (**b**). Sea ice data are based on March values and averaged over a 100-year period centred at the year indicated at the top left of each panel. Sea ice in LOVECLIM is at near-modern extents from 12.4 to 11.6 kyr ago, coincident with the abrupt warming in Greenland temperature in this model (Fig. 4). In CCSM3, however, sea ice continues to extend to the south of Greenland even at the end of the YD (11.2 kyr ago), and Greenland temperatures do not show an abrupt warming. This pattern indicates that sea ice acts as a switch in the models and can abruptly influence temperatures in Greenland.

The abrupt recovery of Greenland temperature from the YD is reproduced in LOVECLIM but not in CCSM3 due to the different behaviour of sea ice in these models (Fig. 4). In LOVECLIM, sea ice expands to the south of Greenland during the YD and then retreats somewhat rapidly (110 years for the onset and 270 years for the termination) to the north on the sudden termination of the meltwater flux at 12.2 kyr ago (Fig. 5). In CCSM3, sea ice continues to cover the oceans adjacent to Greenland throughout the YD recovery (Fig. 5), whereas surface temperature in Greenland increases much more slowly (∼900 years for both the onset and termination) than in LOVECLIM (Fig. 4). In both models, the temperature and precipitation in the northern tropical Atlantic (temperature: 480 years onset, 680 years termination in CCSM and 150 years onset, 600 termination in LOVECLIM; precipitation: 620 years onset, 800 years termination in CCSM and 180 years onset, 420 termination in LOVECLIM) and Indo-Pacific (temperature: no change points detected in CCSM or LOVECLIM; precipitation: 930 years onset, 950 years termination in CCSM and 260 years onset, 450 termination in LOVECLIM) increase more gradually, in agreement with the proxy records. These results suggest that the regional sea ice extent controls the surface air temperature in Greenland^51^. The temperature and precipitation in the northern tropical Atlantic and Indo-Pacific increase more gradually, in agreement with the proxy records. Tropical hydroclimate is likely primarily influenced by hemispheric-scale temperature changes, which require a longer adjustment time than regional sea ice changes^52^. In addition to an increase in the AMOC during the recovery, increasing summer insolation and greenhouse gas concentrations (Fig. 2) also contribute to the slow recovery from the YD in the northern tropics. Decomposition of the climatic responses to these different forcings, based on empirical orthogonal function analysis, suggests that insolation and greenhouse gases may account for approximately two-thirds of the precipitation increase in the tropical Indo-Pacific during the YD recovery (Supplementary Fig. 6), and AMOC changes account for the remaining one-third of the change in Indo-Pacific precipitation.

### Seasonality in the tropical hydroclimate response to the YD

**Figure 6:**
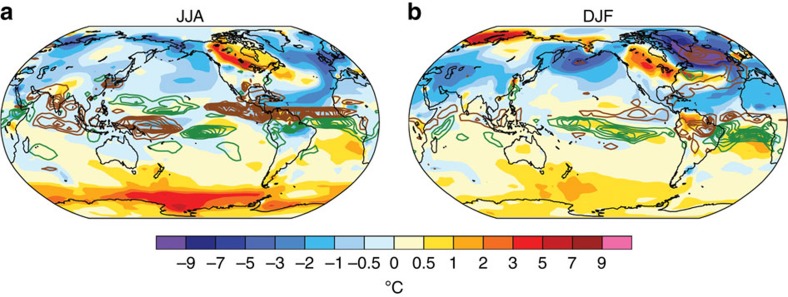
Seasonal distribution of surface temperatures in the CCSM3 model. Temperature (°C, colour shading) and rainfall changes (mm per day, contours) are from before (13.4–13.0 kyr ago) and during (12.4–12.0 kyr ago) the YD for (**a**) JJA and (**b**) DJF as simulated in the CCSM3 TraCE-21ka experiment31. Brown and green contours represent decreased and increased rainfall, respectively, during the YD time period (contour interval: 0.4 mm per day). A general decrease in rainfall is observed over the tropical Indo-Pacific during JJA, although the magnitude of annual rainfall change is only ∼3% (Supplementary Fig. 6), much smaller than the estimate based on the Palawan record (∼25%).

**Figure 7:**
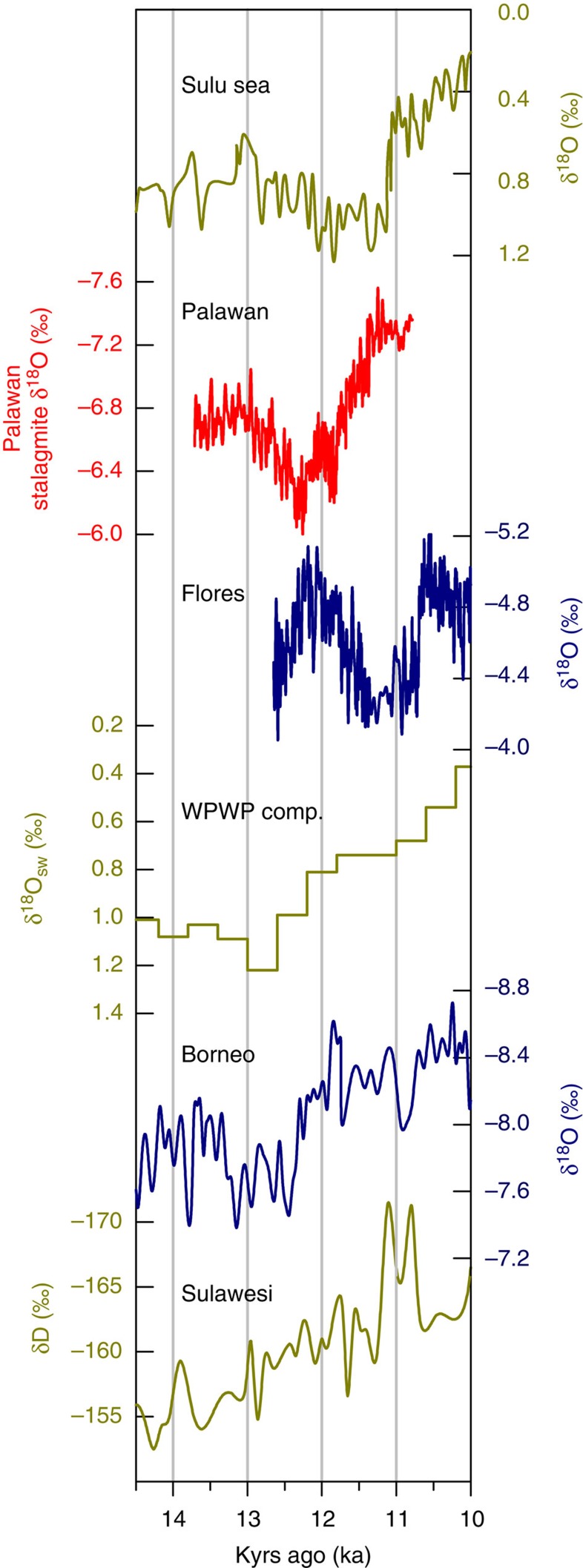
Proxy data for hydroclimate in the WPWP. The record from Palawan, which has a boreal summer bias, shows strong evidence for reduced rainfall, whereas the record from Flores^16^,^70^, which has a boreal winter bias, shows evidence for increased rainfall during the YD. The compilation of marine sediment core seawater δ^18^O_sw_^25^, Borneo stalagmite δ^18^O^15^ and Sulawesi δD_leafwax_^38^ data shows no evidence of a YD signal because these data integrate across the seasons. One of the notable exceptions among marine core δ^18^O_sw_ records is from the Sulu Sea^17^, which formed a more enclosed basin during the YD (Fig. 1, bold coastline) and likely integrated the seasonal precipitation and runoff changes.

In CCSM3, the tropical Atlantic acts as a link that connects AMOC reduction during the YD in the North Atlantic to northern summer monsoon rainfall changes in the Indo-Pacific. During boreal summer, the region of decreased precipitation extends from the northern tropical Atlantic into the eastern tropical Pacific, with alternating signs of precipitation changes in the central (increase) and western Pacific (decrease; Fig. 6). The precipitation decrease over the western Atlantic warm pool in the model, which is also clearly captured in proxy data from the Cariaco Basin^48^, directly stems from the reduction of the AMOC as the ITCZ shifts southward. Reduced precipitation in the western tropical Atlantic intensifies the northeasterly trade winds across Central America, reducing SST and precipitation in the northeastern tropical Pacific^55^. Attendant ocean–atmosphere interactions reorganize the Walker and Hadley circulations^56^,^57^, resulting in decreased precipitation over the western Pacific during boreal summer. Proxy data from sites in the western tropical Pacific that have a pronounced wet season during boreal summer, such as China, the South China Sea and the eastern Philippines (Fig. 1), generally exhibit a decrease in precipitation during the YD (Fig. 7). During boreal winter, tropical precipitation changes simulated by models exhibit a more zonally symmetric pattern relative to that in boreal summer (Fig. 6; Supplementary Fig. 8). Rainfall associated with the ITCZ increases across the Southern Hemisphere in association with the strong cooling in the Northern Hemisphere resulting from the reduced AMOC. The intensified winter westerly jet effectively spreads the cooling in the North Atlantic across the Northern Hemisphere, resulting in a more zonally symmetric response in the tropical hydroclimate^58^, with signs of increased rainfall in the southern tropical Indo-Pacific. These results agree with a similar study of proxy data and climate models studying the response of the western Pacific to changes in the AMOC^27^.

Seasonally dependent changes in precipitation during the YD explain most of the proxy data from the Indo-Pacific (Figs 1 and 7). Rainfall records from sites that are biased towards boreal summer tend to show a decrease in precipitation during the event, whereas rainfall records from sites that are biased towards boreal winter show an increase in precipitation. Sites that represent annual conditions, that is, no seasonal bias, tend to show no response to the YD, which includes both precipitation and surface salinity records. Offsetting changes, that is, a decrease in one season and an increase in the other, during the YD might lead to no change in mean annual conditions, which may be the case in the precipitation records from Borneo^15^ and Sulawesi^38^ and in the marine sediment inferred seawater δ^18^O (δ^18^O_sw_) records (Figs 1 and 7). Many marine sediment δ^18^O_sw_ records^19^,^20^,^23^,^24^,^25^,^31^, a proxy for salinity, integrate the offsetting precipitation changes throughout the year and, hence, do not record a YD signal (Fig. 1b). The marine sediment records in the WPWP that do reveal a hydroclimate response to the YD are in the South China Sea^29^,^30^,^33^, the Sulu Sea^17^ and the eastern Indian Ocean just off of the coast of Sumatra^20^. The hydroclimate signal in the Sulu Sea most likely stems from the fact that this sea, located entirely in the Northern Hemisphere, was more of an enclosed basin when sea level was lower at 12 kyr ago (thick black line in Fig. 1). Therefore, the changes recorded in this sea are likely due to changes in local precipitation and runoff. In addition to local freshwater input, advection by ocean currents likely contributed to the YD salinity signal in the South China Sea and the eastern Indian Ocean marine sediment δ^18^O_sw_ records. Advection of less saline water by ocean currents also explains the marine sediment cores from south of the Equator that indicate drier conditions during the YD^25^,^26^,^27^,^28^. These areas likely did not experience drier conditions locally; rather, the sites recorded the signal of the weaker boreal summer monsoon changes north of the Equator that were then transported southward by the strong currents of the Indonesian throughflow.

## Discussion

Proxy data and model output clearly show how and why changes in the AMOC affect hydroclimate in the western Pacific during the YD, but the difference in timing between the abrupt and the gradual records is difficult to explain. The YD event occurred over decades when viewed through Greenland temperatures due to the ‘rapid retreat of the sea ice cover,' as previously suggested in an early publication on the Greenland ice core record^1^ and supported by the transient climate simulations. However, the transitions into and out of the YD take centuries to complete in tropical hydroclimate records. This temporal disparity is most evident in the difference between records of hydroclimate^48^ and temperature^47^ from the same basin. The most likely factors responsible for tropical rainfall changes on the timescale of centuries are changes in vegetation/land cover, ocean circulation and ice sheets. However, our understanding of how these parameters evolved over time includes large uncertainties due to a lack of data. Although climate models are excellent tools to help elucidate the mechanisms that lead to climate changes, uncertainty in the forcings leads to additional uncertainty in the climate model simulations. A good example of this is the freshwater forcings that are used in the transient climate model simulations: the timing and magnitude of the freshwater added to the North Atlantic markedly differs between the two models, resulting in different temporal evolutions in the simulations (Fig. 4). Uncertainties in forcings are further compounded by model biases. A comparison of output from CCSM3, LOVECLIM and a previous study of idealized freshwater hosing experiments^59^ shows that the details of the precipitation response pattern in the western tropical Pacific are highly model dependent. To better understand changes in the AMOC and global climate, researchers may wish to perform idealized climate model experiments to tease apart different dynamical processes and to compare proxy data and transient climate model simulations from different time periods, such as Heinrich event 1. During Heinrich event 1 (16.8 kyr ago)^60^, the AMOC was reduced for a longer period than during the YD^7^ and large decreases in rainfall were recorded in Borneo^15^, which may reflect a change in both the mean annual rainfall and a change in the seasonal cycle.

Understanding the patterns and mechanisms of the climate response to abrupt climate change events such as the YD has important implications for future climate variability and changes. Studies show that recent changes in the North Atlantic have caused climate changes worldwide^61^,^62^,^63^. Therefore, the YD might be a possible scenario for future changes that might occur under anthropogenic climate forcing. Under global warming, weakening of the AMOC may affect the patterns of regional climate changes, and the YD provides a framework for the changes we might expect to occur. As a modern analogue, analysis of instrumental data for the 20th century suggests that an abrupt cooling of the North Atlantic in the 1960s was accompanied by worldwide shifts in summertime regional climate^61^,^62^, and a recent study indicates that the AMOC is already slowing down^63^. An important implication of the present study is that if there is a marked reduction in the AMOC in the future, the effects will be detectable in northern Atlantic Ocean temperatures within decades, whereas it will take centuries to detect the signal in the tropical hydroclimate. This lag also extends to the recovery period, where the changes in tropical hydroclimate would continue well after the AMOC and Atlantic temperatures recover, thus delaying the return to normal conditions for a large part of the planet. The synthesis of paleoclimate data and the comparison with climate model simulations increases our confidence in the projected effects of AMOC changes on global climate in the future. Last, the late recovery at the end of the YD observed in the new Palawan record and other proxies outside the northern Atlantic Ocean calls for a reassessment of the Greenland ice core records as indicators of how abrupt climate events forced by changes in the AMOC are manifested globally.

## Methods

### Site location

The stalagmite was collected in December 2008 from the Puerto Princesa Subterranean River National Park in Palawan, Philippines (10.2° N, 118.9° E). The main passage of the cave system is an 8.2-km long underground river that empties into the South China Sea. The stalagmite was collected in a chamber (40 × 20 m) located ∼250 m into a side passage. The entrance to the side passage begins ∼200 m from the cave entrance to the sea.

### Stable isotopic composition analysis

Stable isotopic samples were drilled at 1 μm per subsample for the upper 2 cm and 0.5 μm for the lower 16.5 cm of the stalagmite with a computer-assisted micro-milling drilling system (Supplementary Fig. 1). A total of 318 δ^18^O analyses were performed using a Thermo MAT253 isotope ratio mass spectrometer or a Thermo Delta V Plus isotope ratio mass spectrometer with a Kiel IV Carbonate Device, both at the Analytical Laboratory for Paleoclimate Studies at the Jackson School of Geosciences, University of Texas at Austin (repeat measurements of a standard have a long-term 2-sigma analytical precision of 0.12‰). All stalagmite stable isotope values are reported relative to Vienna Pee Dee Belemnite (VPDB) in standard delta notation.

### ^230^Th dating

U–Th age determinations were made on a Thermo NEPTUNE multi-collector inductively coupled mass spectrometer located at the High-Precision Mass Spectrometry and Environment Change Laboratory, National Taiwan University^10^,^64^. Sample weights ranged from 60 to 200 mg, and the average dating error was ±78 years (2*σ*). A Monte Carlo approach simulated age model construction (*N*=10,000) based on a spline fitting using the 2*σ* error of the U–Th dates, allowing for quantification of the temporal error for each δ^18^O measurement (that is, the blue error bars on large isotopic excursions in Fig. 2a and Supplementary Fig. 2). Age corrections were calculated using an estimated atomic ^230^Th/^232^Th ratio of 4±2 p.p.m^10^.

### Climate model simulations

We compared the proxy records with the deglacial climate transition simulated by two climate models of different complexity: the TraCE-21ka experiment with the full physics climate model developed at NCAR (CCSM3)^53^ (http://www.cgd.ucar.edu/ccr/TraCE) and the DG_NS_ experiment with the earth system model of intermediate complexity (LOVECLIM)^54^. CCSM3 and LOVECLIM consist of atmosphere, ocean, sea ice and land surface (with dynamic vegetation) components. LOVECLIM also includes a carbon cycle model. The atmospheric component of CCSM3 is a three-dimensional primitive equation model with a horizontal resolution of T31 (∼3.75°) and 26 vertical levels. The atmospheric model of LOVECLIM is based on quasi-geostrophic equations with a horizontal resolution of T21 (∼5.63°) and three vertical levels. These models are forced with temporally varying greenhouse gas concentrations, insolation, ice-sheet topography and meltwater fluxes from 22 kyr ago to the present (1990) for CCSM3 and from 21 to 10 kyr ago for LOVECLIM. See the references for further details on the forcing and model configurations.

We compared the tropical Indo-Pacific rainfall simulated in the CCSM3 TraCE-21ka experiment with instrumental data and the Palawan record. The climatological seasonal cycle before the YD in CCSM3 agreed well with instrumental data (Supplementary Fig. 6). However, the magnitude of the YD precipitation decrease was small in CCSM3, amounting to ∼3% of the total annual rainfall (Supplementary Fig. 6). The Palawan record implies a rainfall decrease of 0.5 m per year on average for the decadal-scale data or an ∼25% reduction in annual rainfall, highlighting a substantial difference in the quantitative estimates between the model output and the proxy data.

### Data archiving

The ^230^Th age-depth models and δ^18^O data from the Palawan stalagmite are archived at the NOAA World Data Center for Paleoclimatology at https://www.ncdc.noaa.gov/data-access/paleoclimatology-data/datasets.

## Additional information

**How to cite this article:** Partin, J. W. *et al*. Gradual onset and recovery of the Younger Dryas abrupt climate event in the tropics. *Nat. Commun.* 6:8061 doi: 10.1038/ncomms9061 (2015).

## Supplementary Material

Supplementary Information Supplementary Figures 1-8, Supplementary Tables 1-2 and Supplementary References
