# Supplementary material for: Gradual onset and recovery of the Younger Dryas abrupt climate event in the tropics
Source: Nat Commun. 2015 Sep 2;6:8061. doi: 10.1038/ncomms9061 (PMC4569703; doi:10.1038/ncomms9061)
Supplement: Supplementary Information — Supplementary Figures 1-8, Supplementary Tables 1-2 and Supplementary References [file ncomms9061-s1.pdf]

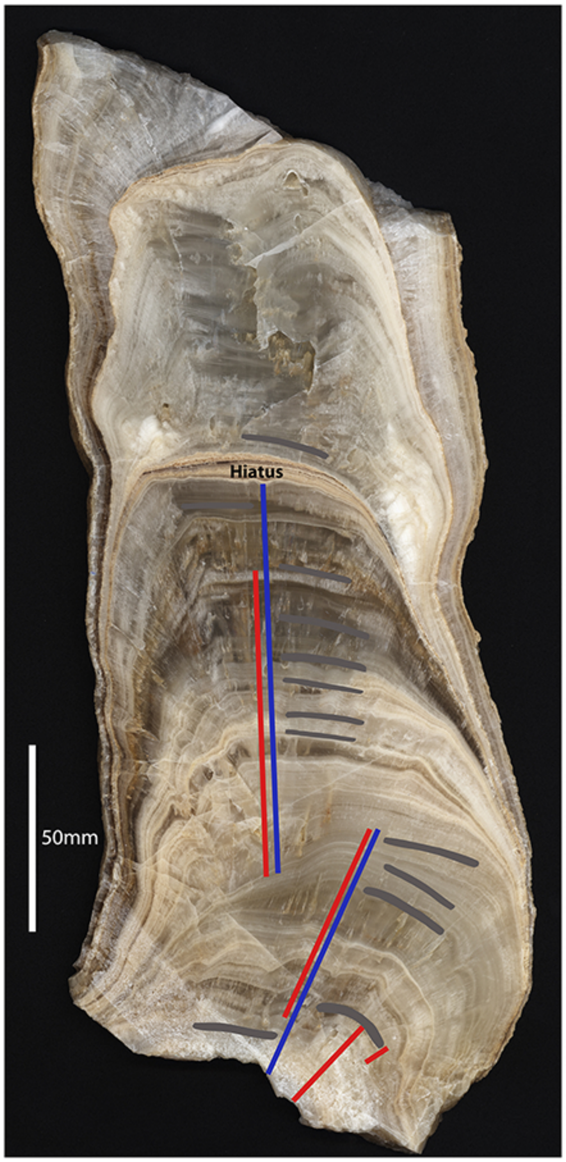

Supplementary Figure 1 | Scan of Stalagmite SR02 with drill paths. The dark blue path was drilled at 1mm resolution, and the red path was drilled at 0.5 mm resolution for $\delta^{18}O$ analyses (see Supplementary Figure 3 for $\delta^{18}O$ data). Dark grey markers along a growth band indicate where sub-samples were drilled for U-Th dating. Some of the U-Th had clear detrial Th contamination and were not used. The age above the dark brown layers labeled 'Hiatus' is 9995 yrs BP and the age below is 10967 yrs BP, providing clear evidence for the hiatus in growth.

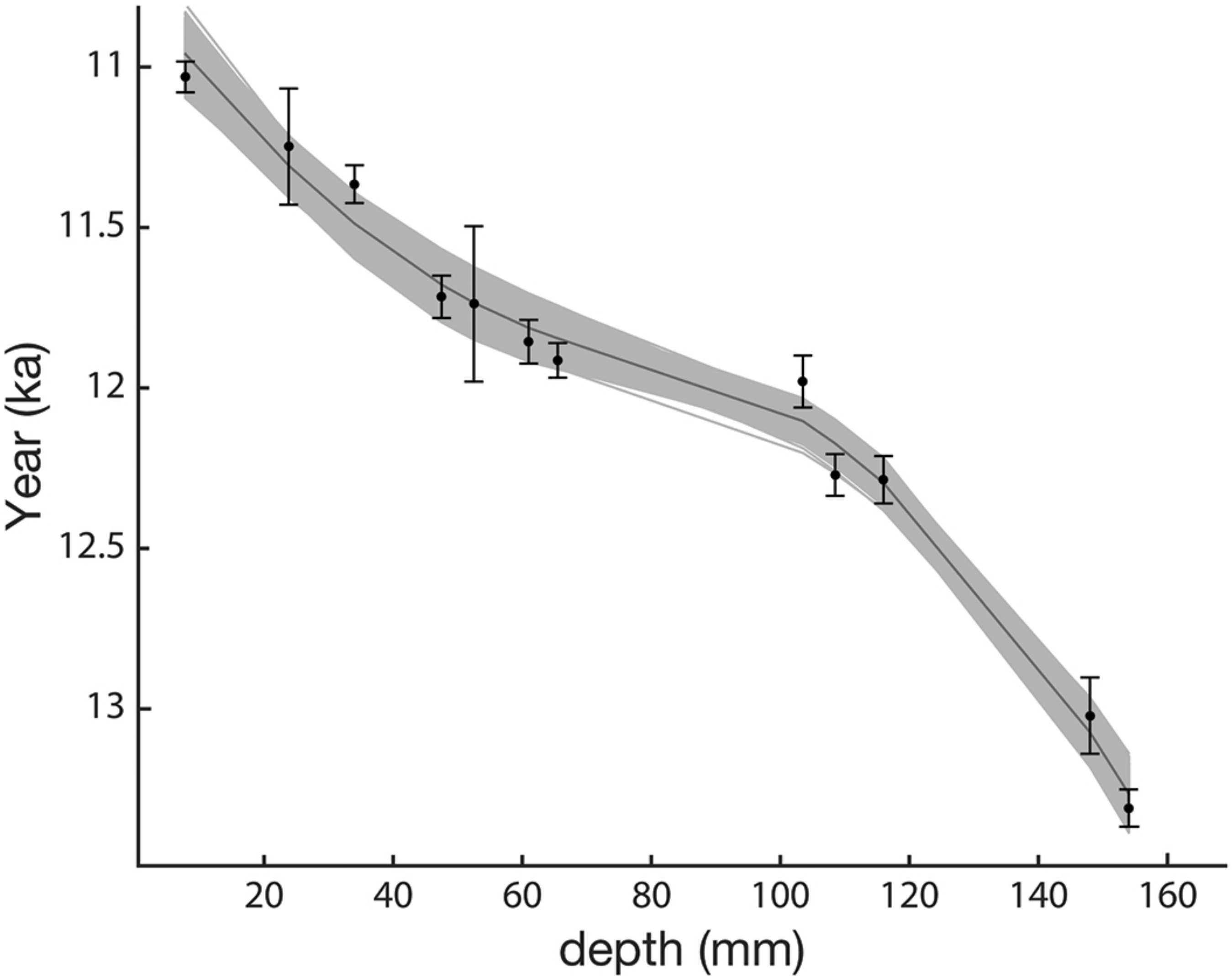

Supplementary Figure 2 | Age model for stalagmite SR02 based on 12 U-Th dates. The dark curve in the middle of the grey spread represents the median age model. The 10,000 Monte Carlo iterations of alternate age models are plotted in grey.

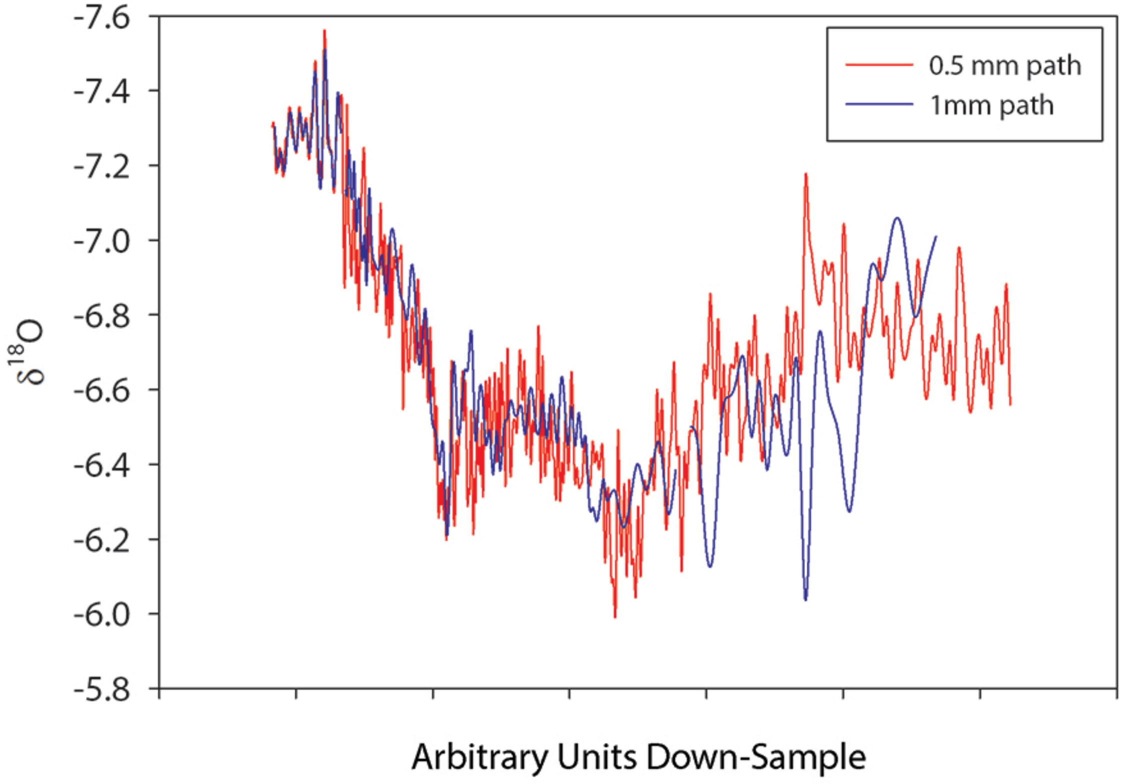

Supplementary Figure 3 | Stalagmite $\delta^{18}O$ from stalagmite SR02 in two parallel paths. Onepath was sub-sampled at 0.5 mm intervals (red), and one was sub-sampled at 1mm intervals (blue) (see Supplementary Fig. 1 for locations). Overall, there is good replication between the paths, providing positive support for passing the 'Hendy test'. At the bottom of the stalagmite, there is some disagreement due to the fact that the 1mm path was drilled off the central growth axis (Supplementary Fig. 1). Therefore the mismatch is not due to kinetic effects, but rather that the blue path is integrating time differently.

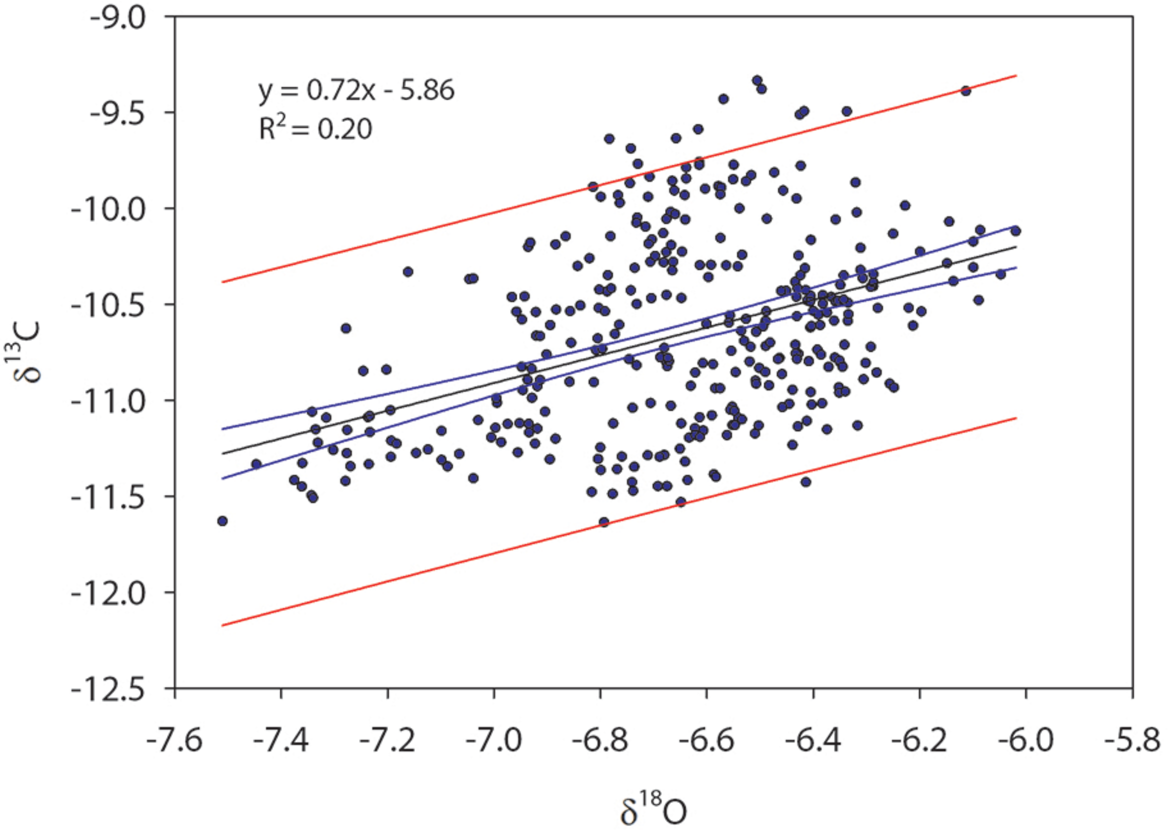

Supplementary Figure 4 | Stalagmite $\delta^{18}O$ versus $\delta^{13}C$ as part of the 'Hendy test'. The low correlation coefficient ($R^2 = 0.20$) between $\delta^{18}O$ versus $\delta^{13}C$ provides further confirmation that the stalagmite precipitated in isotopic equilibrium with cave dripwater. Blue and red curves represent the 95% confidence and prediction intervals of the linear regression (black curve), respectively.

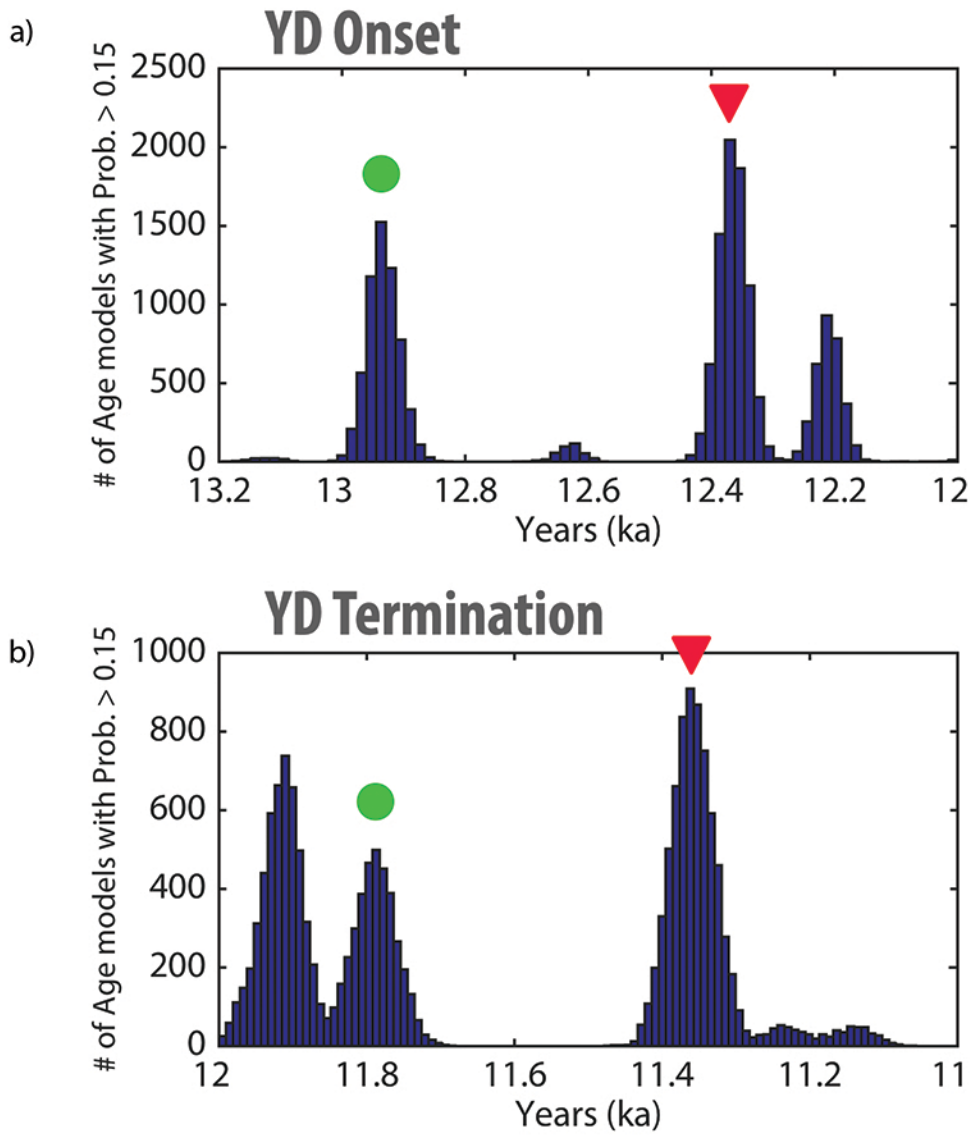

Supplementary Figure 5 | Timing of the Younger Dryas in the Palawan stalagmite δ^{18}O record. The onset (a) and termination (b) of the Younger Dryas (YD) are described by both the initiation and completion of each. The green cirle indicates the initiation of either the onset or termination, and the red triangle indicates the completion (as in Fig. 3). The change point calculation (Ruggieri, 2013) was made on 5000 different age models of the stalagmite timeseries to get a distribution of timings for the transitions. The width of the distribution is used for the error bar in Fig. 3.

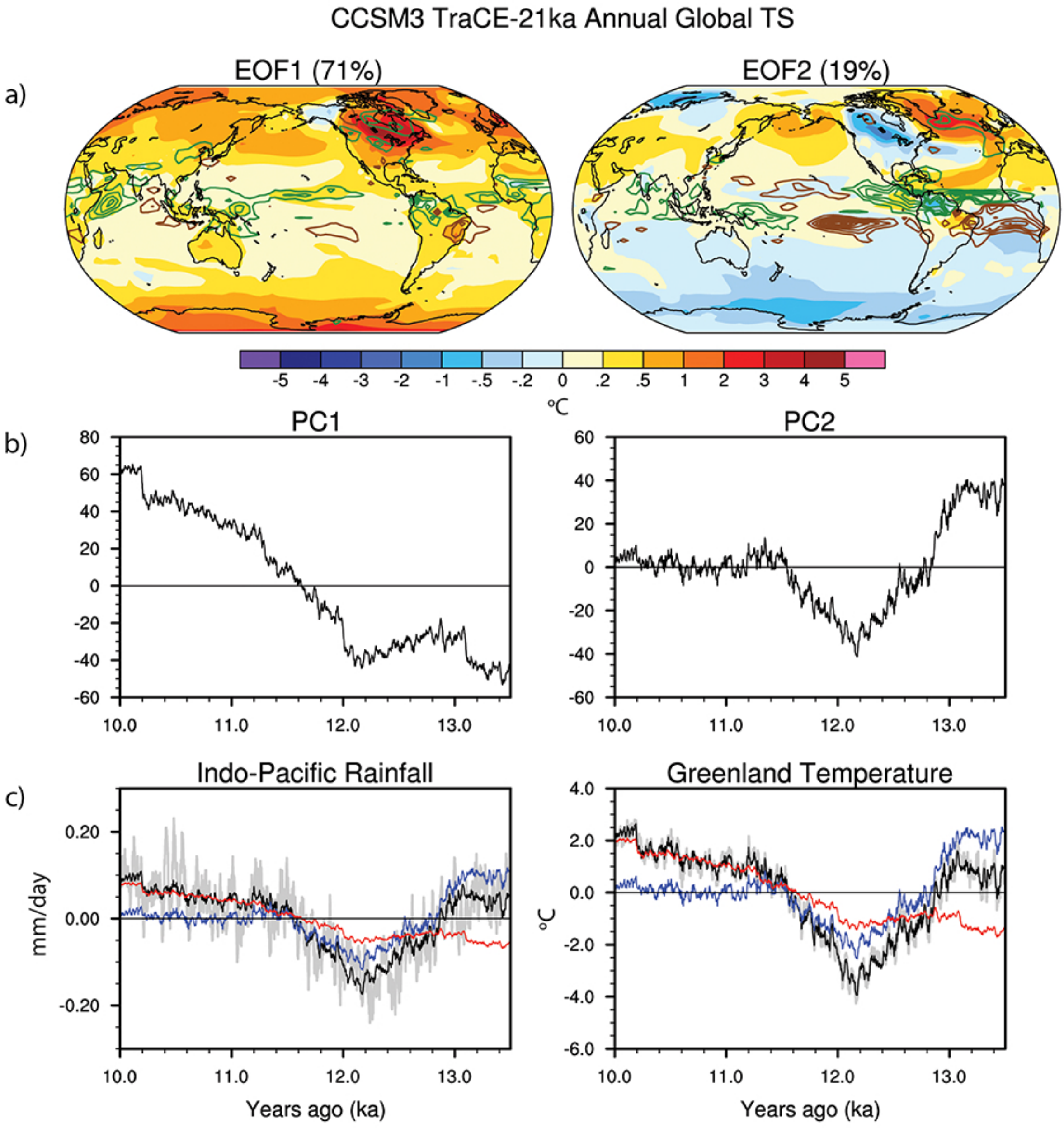

Supplementary Figure 6 | Decomposition of global climate response to greenhouse gas and meltwater forcings in the CCSM3 TraCE-21ka experiment during 13.5-10.0 ka. a) The two leading EOF patterns of global surface temperature (°C, shading). Global rainfall is also regressed onto the leading PCs (negative/positive contours in brown/green at intervals of 0.2 mm/day). The EOF1 and EOF2 account for 71% and 19% of the total variance, respectively. The data is averaged annually and smoothed with a 21-year running mean filter prior to the analysis. b) Time series of the PC1 and PC2. c) Time series of the tropical Indo-Pacific rainfall and Greenland temperature (gray) and contributions from EOF1 (red), EOF2 (blue), and their sum (black). The EOF1 exhibits a global warming pattern and the PC1 time series closely resembles the temporal evolution of CO_2 (and summer insolation in the NH, see Figure 2). The EOF2 shows a bipolar seesaw pattern between the two hemispheres and the PC2 time series follows the meltwater forcing prescribed in this model. Greenhouse gas and meltwater forcings together explain large part of the temporal evolution of tropical Indo-Pacific rainfall and Greenland temperature as evidenced by the similarity of gray and black curves in panel (c). The CO_2 increase after 12 ka contributes to about one third of the recov ery from the YD in both the tropical Indo-Pacific rainfall and Greenland temperature.

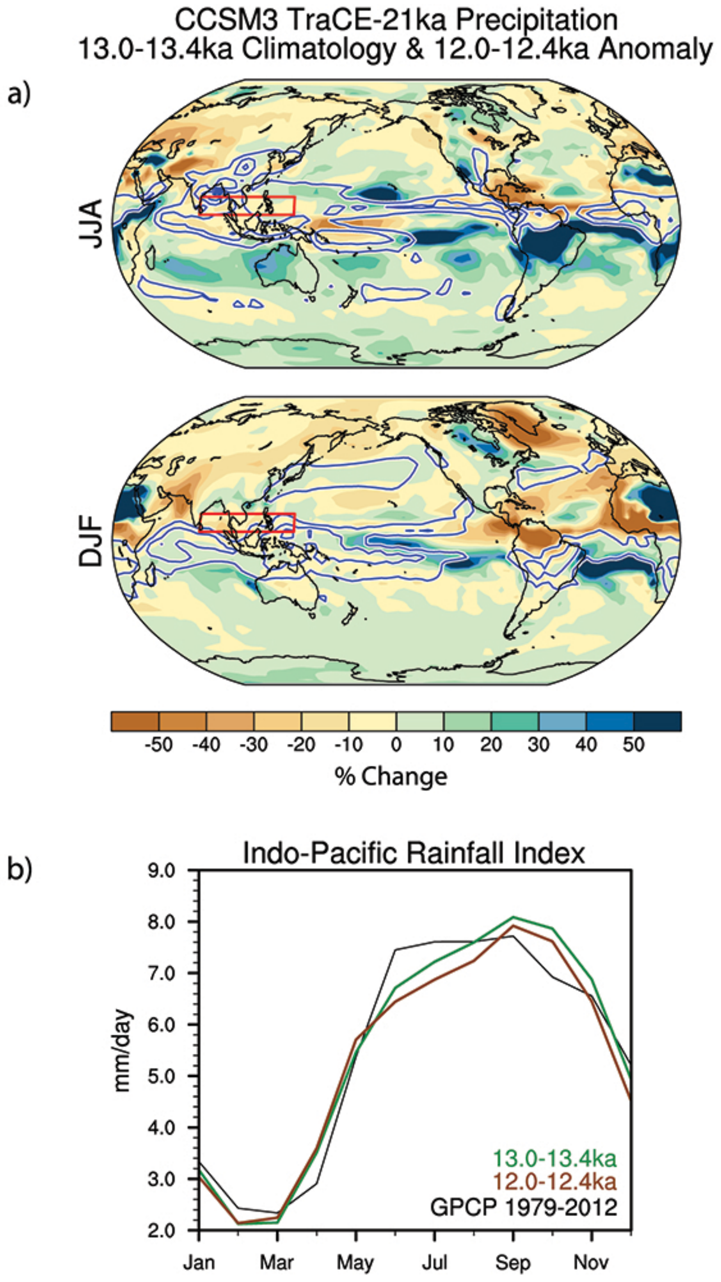

Supplementary Figure 7 | Rainfall climatology and anomalies for seasons JJA and DJF in the CCSM3 TraCE-21ka experiment. a) Global maps of rainfall climatology before the YD (13.4-13.0 ka; blue contours at intervals of 4 mm/day) and anomalies during the YD (12.4-12.0 ka) as a percent change from climatology (shading). The red box marks the northern tropical Indo-Pacific region where the model rainfall output was averaged and plotted in Fig. 2 and in panel (b). b) The annual cycle of rainfall in the tropical Indo-Pacific before (13.4-13.0 ka, green) and during (12.4-12.0 ka, brown) the YD. While the model output shows a decrease in rainfall during the YD, the difference in rainfall during the YD amounts to a reduction of ~3% of annual rainfall. The Palawan record implies a rainfall decrease of 0.5 m/year or an ~25% reduction in annual rainfall highlighting a large difference in the quantitative estimates between the model output and the proxy data.

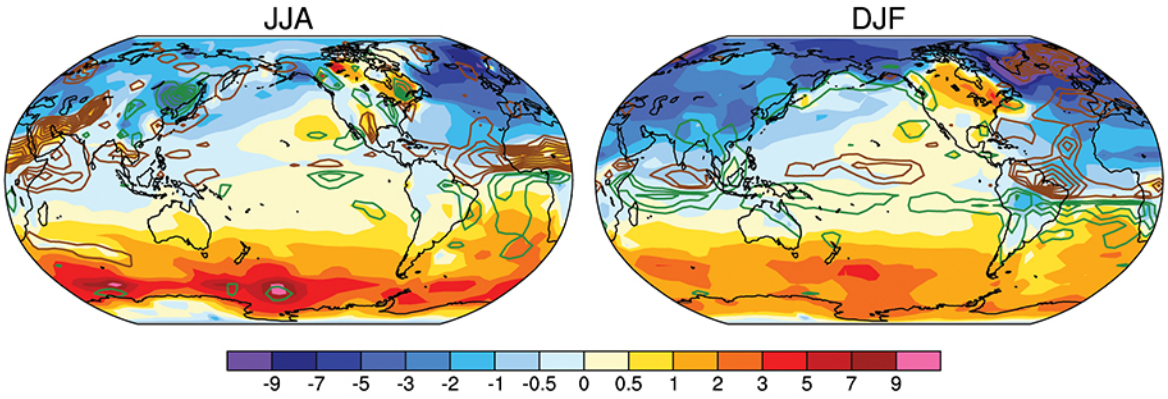

Supplementary Fig. 8 | Seasonal distribution of surface temperature and rainfall as simulated in the LOVECLIM DGNS experiment. Surface temperature (°C, shading) and rainfall changes (mm/day, contours) are from before (13.6-13.2 ka) and during (12.8-12.4 ka) the YD. Brown (green) contours indicate rainfall reduction (increase) during the YD time period (contour interval: 0.4 mm/day). There is a general decrease in rainfall over the northern tropical Indo-Pacific during JJA while the DJF pattern is characterized by southward shift of the ITCZ across all three basins

Supplementary Table 1 | List of studies used in Figure 1

Temperature Proxies

Lat-Long	General Location	Proxy	YD Response	Citation #
(27°49.20N, 126°58.70E)	South China Sea	Mg/Ca	Cooling	1
(20° 07.0' N, 117° 23.0'E)	South China Sea	Alkenone	Cooling	2
(14.82°N, 123.49°E)	South China Sea	Mg/Ca	Cooling	3
(8.8°N, 121.3°E)	Sulu Sea	Mg/Ca	None	4
(8°43.73'N, 109°52.17'E)	South China Sea	Alkenone	Cooling	5
(8° 30.4'N, 112° 19.9'E)	South China Sea	Mg/Ca	None	6
(6°27' N, 125°50' E)	Mindanao Sea	Mg/Ca	None	7
(6° 38.12 N, 113° 24.56 E)	South China Sea	Alkenone	Cooling	8
(5°39'N, 110°39'E)	South China Sea	Alkenone	Cooling	9
(3°31'N, 96°19' E)	E. Indian Ocean	Mg/Ca	None	10
(2° 02' N, 141° 45'E)	Western Pacfic	Mg/Ca	None	11
(1° 25' N, 146° 14'E)	Western Pacfic	Mg/Ca	None	12
(0°47'S, 99°04' E)	E. Indian Ocean	Mg/Ca	None	10
(1°29.67'S, 100°07.68' E)	E. Indian Ocean	Mg/Ca	None	13
(13°5' S, 121°47' E)	Timor Sea	Mg/Ca	None	14
(3° 34' S, 119° 23' E)	S. Makassar Strait	Mg/Ca	None	15
(4° 41.33' S, 117° 54.17' E)	S. Makassar Strait	Mg/Ca	None	16
(5°0' S, 133° 26'E)	Banda Sea	Mg/Ca	None	7
(5°56'S, 103°15' E)	E. Indian Ocean	Mg/Ca	None	13
(9°36' S, 120°55' E)	Savu/Timor Sea	Mg/Ca	None	17
(9°39' S, 118°20' E)	Indonesian archipelago/Timor Sea/Indian Ocean	Mg/Ca	None	18

Hydrologic Proxies

Lat-Long	General Location	Proxy	YD Response	Citation #
(32°30N, 119°10'E)	China	$\delta^{18}O$	Summer Monsoon	19
(27°49.20N, 126°58.70E)	South China Sea	$\delta^{18}O_{sw}$	Drier	1
(25°17'N, 108°5'E)	China	$\delta^{18}O$	Summer Monsoon	20
(21°9'N, 110°17'E)	China	Magnetic Susceptibility	Wetter Boreal Winter Monsoon	21
(20° 07.0' N, 117° 23.0'E)	South China Sea	$\delta^{18}O_{sw}$	Drier	2
(14.82°N, 123.49°E)	South China Sea	$\delta^{18}O_{sw}$	None	3
(8.8°N, 121.3°E)	Sulu Sea	$\delta^{18}O_{sw}$	Drier	4
(6° 38.12 N, 113° 24.56 E)	South China Sea	$\delta^{18}O_{sw}$	Drier	8
(6°27' N, 125°50' E)	Mindanao Sea	$\delta^{18}O_{sw}$	None	7
(4°12'N, 114°56'E)	N. Borneo	$\delta^{18}O$	None	22
(3°31'N, 96°19' E)	E. Indian Ocean	$\delta^{18}O_{sw}$	None	10
(1°09'N, 98°04' E)	E. Indian Ocean	$\delta^{18}O$	None	10
(0°47'S, 99°04' E)	E. Indian Ocean	$\delta^{18}O_{sw}$	None	10
(1°29.67'S, 100°07.68' E)	E. Indian Ocean	$\delta^{18}O_{sw}$	None	13
(3° 34' S, 119° 23' E)	S. Makassar Strait	$\delta^{18}O_{sw}$	Drier	15
(3.566°S, 119.383°E)	Sulawesi/Makassar Strait	δD	None	23
(4° 41.33' S, 117° 54.17' E)	S. Makassar Strait	$\delta^{18}O_{sw}$	Drier	16
(5° 00.18' S, 133° 26'E)	Banda Sea	$\delta^{18}O_{sw}$	None	7
(5°56'S, 103°15' E)	E. Indian Ocean	$\delta^{18}O_{sw}$	None	13
(8°32' S, 120°26' E)	Flores, Indonesia	$\delta^{18}O$	Wetter Austral Summer	24,25
(9°36' S, 120°55' E)	Savu/Timor Sea	$\delta^{18}O_{sw}$	Drier	17
(9°39' S, 118°20' E)	Indonesian archipelago/Timor Sea/Indian Ocean	$\delta^{18}O_{sw}$	Drier	18
(13°5' S, 121°47' E)	Timor Sea	$\delta^{18}O_{sw}$	None	14

Supplementary Table 2 | U-Th dates

Depth (mm)	Date of Chemistry	Weight (g)	^{238}U (ppb)	^{232}Th (ppt)	$\delta^{234}U$ measured[a]	$[^{230}Th/^{238}U]$ activity[c]	$[^{230}Th/^{232}Th]$ ppm[d]	Age uncorrected	Age corrected [c,e]	Age Years BP, before 1950	$\delta^{234}U_{initial}$ corrected[b]
above hiatus	Aug 2012	0.08210	423.94 ± 0.59	755.2 ± 5.8	243.7 ± 2.0	0.10895 ± 0.00045	1008 ± 9	9,968 ± 46	9,931 ± 50	9,995 ± 50	250.6 ± 2.1
4	Aug 2012	0.06720	498.42 ± 0.85	347.5 ± 7	260.4 ± 2.7	0.12113 ± 0.00042	2865 ± 58	10,982 ± 47	10,967 ± 48	11,031 ± 48	268.6 ± 2.8
7	Dec 2014	0.08609	421.99 ± 0.94	1039.5 ± 6	254.7 ± 3.0	0.12310 ± 0.00052	824 ± 5	11,222 ± 57	11,171 ± 63	11,248 ± 63	262.9 ± 3.1
21	Aug 2012	0.09940	587.9 ± 1.1	7043 ± 31	264.1 ± 3.0	0.1263 ± 0.0013	173.8 ± 2.0	11,435 ± 131	11,186 ± 181	11,365 ± 181	272.6 ± 3.1
33.5	Aug 2012	0.07460	563.2 ± 1.2	168.1 ± 6.2	250.2 ± 3.5	0.12355 ± 0.00051	6827 ± 255	11,309 ± 59	11,303 ± 59	11,716 ± 59	258.3 ± 3.6
43.5	Dec 2014	0.06568	452.9 ± 0.9	1179.4 ± 7.3	246.9 ± 2.9	0.12734 ± 0.00055	806 ± 6	11,707 ± 61	11,652 ± 66	11,738 ± 66	255.1 ± 3.0
58	Dec 2014	0.10063	461.0 ± 1.0	1412.1 ± 5.1	245.9 ± 2.6	0.12878 ± 0.00055	693 ± 4	11,857 ± 59	11,792 ± 68	11,856 ± 68	254.2 ± 2.7
63.5	Dec 2014	0.14229	504.6 ± 1.1	856.9 ± 3.5	258.4 ± 2.6	0.13039 ± 0.00046	1266 ± 6	11,885 ± 51	11,850 ± 54	11,914 ± 54	267.2 ± 2.7
99.5	Aug 2012	0.09250	373.05 ± 0.72	1045.0 ± 5.8	260.8 ± 3.1	0.13160 ± 0.00072	774.6 ± 5.8	11,977 ± 76	11,918 ± 81	11,980 ± 81	269.7 ± 3.2
109.5	Dec 2014	0.10964	484.02 ± 0.78	1300.3 ± 4.6	280.0 ± 2.3	0.13665 ± 0.00056	838.7 ± 4.4	12,262 ± 58	12,207 ± 65	12,271 ± 65	289.8 ± 2.4
117	Aug 2012	0.08260	534.0 ± 1.2	1038.6 ± 6.4	272.5 ± 3.5	0.13586 ± 0.00064	1151.8 ± 8.6	12,264 ± 71	12,224 ± 74	12,286 ± 74	282.0 ± 3.6
152	Aug 2012	0.06180	542.9 ± 1.4	2826 ± 13	289.5 ± 3.8	0.1462 ± 0.0010	463.1 ± 3.7	13,065 ± 107	12,959 ± 119	13,021 ± 119	300.3 ± 3.9
169	May 2013	0.2014	547.33 ± 0.98	960.3 ± 2.8	275.4 ± 2.4	0.1469 ± 0.0005	1380.0 ± 5.7	13,283 ± 55	13,247 ± 58	13,309 ± 58	285.9 ± 2.5

Chemistry was performed on Aug. 2012, May 2013, and Dec 2014 (ref. 26).

Instrumental analysis on MC-ICP-MS[27].

Analytical errors are 2σ of the mean.

[a] $[^{238}U] = [^{235}U] \times 137.818$ (±0.65‰) (Hiess et al., 2012); $\delta^{234}U = ([^{234}U/^{238}U]_{activity} - 1) \times 1000$.

[b] $\delta^{234}U_{initial}$ corrected was calculated based on ^{230}Th age (T), i.e., $\delta^{234}U_{initial} = \delta^{234}U_{measured}\ X\ e^{l234*T}$, and T is corrected age.

[c] $[^{230}Th/^{238}U]_{activity} = 1 - e^{-l230T} + (d^{234}U_{measured}/1000)[l_{230}/(l_{230} - l_{234})](1 - e^{-(l230 - l234)T})$, where T is the age.

Decay constants are 9.1705×10^{-6} yr^{-1} for ^{230}Th, 2.8221×10^{-6} yr^{-1} for ^{234}U (ref. 28), and 1.55125×10^{-10} yr^{-1} for ^{238}U (ref. 29).

[d] The degree of detrital ^{230}Th contamination is indicated by the $[^{230}Th/^{232}Th]$ atomic ratio instead of the activity ratio.

[e] Age corrections for samples were calculated using an estimated atomic $^{230}Th/^{232}Th$ ratio of 4 ± 2 ppm

Those are the values for a material at secular equilibrium, with the crustal $^{232}Th/^{238}U$ value of 3.8. The errors are arbitrarily assumed to be 50%.

Supplementary References

1. Sun, Y., Oppo, D. W., Xiang, R., Liu, W. & Gao, S. Last deglaciation in the Okinawa Trough: Subtropical northwest Pacific link to Northern Hemisphere and tropical climate. *Paleoceanography* **20,** PA4005 (2005).
2. Wang, L. *et al.* East Asian monsoon climate during the Late Pleistocene: high-resolution sediment records from the South China Sea. *Marine Geology* **156,** 245–284 (1999).
3. Dang, H., Jian, Z., Bassinot, F., Qiao, P. & Cheng, X. Decoupled Holocene variability in surface and thermocline water temperatures of the Indo-Pacific Warm Pool. *Geophys. Res. Lett.* **39,** L01701 (2012).
4. Rosenthal, Y. The amplitude and phasing of climate change during the last deglaciation in the Sulu Sea, western equatorial Pacific. *Geophys. Res. Lett.* **30,** 1428 (2003).
5. Zhao, M., Huang, C.-Y., Wang, C.-C. & Wei, G. A millennial-scale U37K′ sea-surface temperature record from the South China Sea (8°N) over the last 150 kyr: Monsoon and sea-level influence. *Palaeogeography, Palaeoclimatology, Palaeoecology* **236,** 39–55 (2006).
6. Pelejero, C., Grimalt, J. O., Heilig, S., Kienast, M. & Wang, L. High-resolution UK37 temperature reconstructions in the South China Sea over the past 220 kyr. *Paleoceanography* **14,** 224–231 (1999).
7. Stott, L. *et al.* Decline of surface temperature and salinity in the western tropical Pacific Ocean in the Holocene epoch. *Nature* **431,** 56–59 (2004).
8. Steinke, S. *et al.* Proxy dependence of the temporal pattern of deglacial warming in the tropical South China Sea: toward resolving seasonality. *Quaternary Science Reviews* **27,** 688–700 (2008).
9. Kienast, M., Steinke, S., Stattegger, K. & Calvert, S. E. Synchronous tropical South China Sea SST change and Greenland warming during deglaciation. *Science* **291,** 2132–2134 (2001).
10. Mohtadi, M. *et al.* North Atlantic forcing of tropical Indian Ocean climate. *Nature* **509,** 76–80 (2014).
11. de Garidel-Thoron, T., Rosenthal, Y., Bassinot, F. & Beaufort, L. Stable sea surface temperatures in the western Pacific warm pool over the past 1.75 million years. *Nature* **433,** 294–298 (2005).
12. de Garidel-Thoron, T. *et al.* A multiproxy assessment of the western equatorial Pacific hydrography during the last 30 kyr. *Paleoceanography* **22,** PA3204 (2007).
13. Mohtadi, M., Steinke, S., Lückge, A., Groeneveld, J. & Hathorne, E. C. Glacial to Holocene surface hydrography of the tropical eastern Indian Ocean. *Earth and Planetary Science Letters* **292,** 89–97 (2010).
14. Xu, J., Holbourn, A., Kuhnt, W., Jian, Z. & Kawamura, H. Changes in the thermocline structure of the Indonesian outflow during Terminations I and II. *Earth and Planetary Science Letters* **273,** 152–162 (2008).
15. Linsley, B. K., Rosenthal, Y. & Oppo, D. W. Holocene evolution of the

Indonesian throughflow and the western Pacific warm pool. *Nature Geoscience* **3,** 578–583 (2010).
16. Visser, K., Thunell, R. & Stott, L. Magnitude and timing of temperature change in the Indo-Pacific warm pool during deglaciation. *Nature* **421,** 152–155 (2003).
17. Gibbons, F. T. *et al.* Deglacial δ18O and hydrologic variability in the tropical Pacific and Indian Oceans. *Earth and Planetary Science Letters* **387,** 240–251 (2014).
18. Levi, C. *et al.* Low-latitude hydrological cycle and rapid climate changes during the last deglaciation. *Geochem. Geophys. Geosyst.* **8,** Q05N12 (2007).
19. Wang, Y. J. A High-Resolution Absolute-Dated Late Pleistocene Monsoon Record from Hulu Cave, China. *Science* **294,** 2345–2348 (2001).
20. Dykoski, C. A. *et al.* A high-resolution, absolute-dated Holocene and deglacial Asian monsoon record from Dongge Cave, China. *Earth and Planetary Science Letters* **233,** 71–86 (2005).
21. Yancheva, G. *et al.* Influence of the intertropical convergence zone on the East Asian monsoon. *Nature* **445,** 74–77 (2007).
22. Partin, J. W., Cobb, K. M., Adkins, J. F., Clark, B. & Fernandez, D. P. Millennial-scale trends in west Pacific warm pool hydrology since the Last Glacial Maximum. *Nature* **449,** 452–455 (2007).
23. Tierney, J. E. *et al.* The influence of Indian Ocean atmospheric circulation on Warm Pool hydroclimate during the Holocene epoch. *J. Geophys. Res.* **117,** D19108 (2012).
24. Griffiths, M. L. *et al.* Increasing Australian--Indonesian monsoon rainfall linked to early Holocene sea-level rise. *Nature Geoscience* **2,** 636–639 (2009).
25. Ayliffe, L. K. *et al.* Rapid interhemispheric climate links via the Australasian monsoon during the last deglaciation. *Nature Communications* **4,** 1–6 (2013).
26. Shen, C.-C. *et al.* Measurement of Attogram Quantities of 231Pa in Dissolved and Particulate Fractions of Seawater by Isotope Dilution Thermal Ionization Mass Spectroscopy. *Anal. Chem.* **75,** 1075–1079 (2003).
27. Shen, C.-C. *et al.* High-precision and high-resolution carbonate. *Geochimica et Cosmochimica Acta* **99,** 71–86 (2012).
28. Cheng, H. *et al.* Improvements in 230Th dating, 230Th and 234U half-life values, and U–Th isotopic measurements by multi-collector inductively coupled plasma mass spectrometry. *Earth and Planetary Science Letters* **371-372,** 82–91 (2013).
29. Jaffey, A. H., Flynn, K. F., Glendenin, L. E., Bentley, W. C. & Essling, A. M. Precision measurement of half-lives and specific activities of 235U and 238U. *Physical Review C* **4,** 1889–1906 (1971).
